# Pharmaceuticals: An Outbreak of New Sources of Avian Flu Drug

**Published:** 2006-08

**Authors:** Cynthia Washam

Worldwide, 228 people have been infected with H5N1 avian influenza, largely through exposure to sick birds; of these, more than half have died. Although only limited human-to-human transmission has been confirmed, scientists fear a worldwide pandemic could erupt if the virus mutates to a highly pathogenic form that humans can efficiently pass among themselve. Now scientists are finding faster, cheaper ways to produce more of the only drug proven capable of combating avian flu.

Tamiflu (oseltamivir phosphate) reduces flu mortality by inhibiting the virus from spreading among cells. For several years Roche Pharmaceuticals has made the drug with shikimic acid from the pod of the star anise tree, a native of Asia. Extracting the acid is slow and expensive, but productive enough to meet the demand for regular seasonal flu. Recent “shortages” occurred when countries started stockpiling the drug in anticipation of a potential pandemic.

In the 17 May 2006 *Journal of the American Chemical Society*, two separate teams describe new methods for synthesizing oseltamivir phosphate without using shikimic acid. “We came up with a very efficient route,” says Harvard University chemist Elias Corey of his petrochemical-based method. “The yield is twice as much as with the present process.” In the other new method, Masakatsu Shibasaki and colleagues at the University of Tokyo use 1,4-cyclohexadiene, a benzene derivative, as a catalyst.

Other researchers are taking another tack: finding new sources of shikimic acid. Chemistry professor Thomas Poon of Claremont McKenna College has extracted the acid from the seeds of sweetgum trees, while Canada-based Biolyse Pharma found a source in the needles of discarded pine, fir, and spruce Christmas trees. Neither of these methods has been published.

Roche has significantly expanded its Tamiflu production capacity over the past several years, and will be able to produce up to 400 million treatment courses annually by the end of 2006—a more than 10-fold increase over 2004 capacity. Production is getting a boost in part as Roche replaces most of the star anise extraction with *Escherichia coli* fermentation. The bacteria produce shikimic acid quickly and cheaply from glucose. Roche and its partners plan to substantially increase their fermentation capacities over the coming years.

Roche spokesman Terence Hurley wouldn’t say whether the company anticipates adopting any other new methods. He did point out that a new process would require approval of the FDA and its foreign counterparts.

If Roche doesn’t use his technique, Corey hopes another manufacturer does. This could happen despite Roche’s patent rights—if it ever does come down to a human pandemic, the 2001 Doha Declaration of the World Trade Organization states that countries facing a public health crisis may grant licenses for production of patented drugs.

## Figures and Tables

**Figure:**
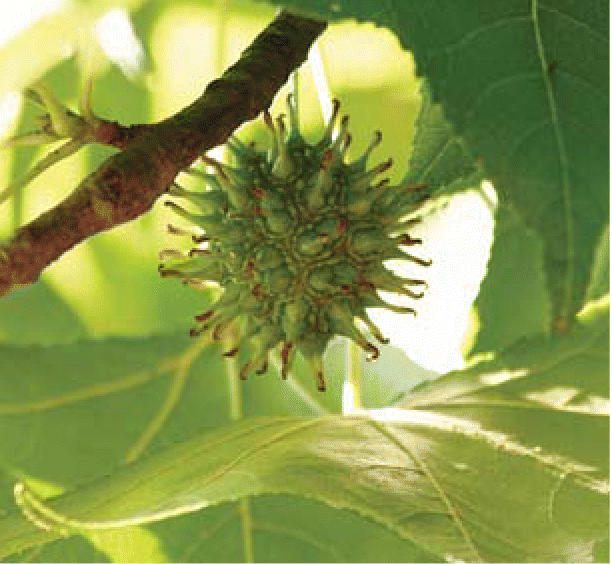
Sweetgum surprise Researchers are finding new sources of shikimic acid.

